# Supplementary material for: Plastid phylogenomics reveals evolutionary relationships in the mycoheterotrophic orchid genus Dipodium and provides insights into plastid gene degeneration
Source: Front Plant Sci. 2024 Jun 13;15:1388537. doi: 10.3389/fpls.2024.1388537 (PMC11210000; doi:10.3389/fpls.2024.1388537)
Supplement: Supplementary Material 1 — Details of samples included in phylogenetic analysis and divergence-time estimations. [file DataSheet_1.pdf]

frontiers

Supplementary Material 1

Plastid phylogenomics reveals evolutionary relationships in the mycoheterotrophic orchid genus *Dipodium* and provides insights into plastid gene degeneration

Stephanie Goedderz*, Mark A. Clements, Stephen J. Bent, James A. Nicholls, Vidushi S. Patel, Darren M. Crayn, Philipp M. Schlüter, Katharina Nargar*

*** Correspondence:**
Stephanie Goedderz: stephanie.goedderz@jcu.edu.au
Katharina Nargar: katharina.nargar@csiro.au

Table S1.1: **Taxa included in phylogenetic analysis and divergence-time estimations.** Taxonomic concepts follow CHAH (2023) https://biodiversity.org.au/nsl/services/search/taxonomy for Australian taxa and WFO (2023): http://www.worldfloraonline.org for all other regions (accessed in May 2023) except for taxa with asterisks for which a synonym was used (shown in square brackets).

**Species**	**Subtribe**	**Tribe**	**Subfamily**	**NCBI/ ENA no.**	**DNA no.**	**Included in divergence-time analysis**
Aa paleacea (Kunth) Rchb.f.	Cranichidiinae	Cranichideae	Orchidoideae	Givnish *et al.* (2015)	-	yes
Acriopsis emarginata D.L.Jones & M.A.Clem.	Cymbidiinae	Cymbidieae	Epidendroideae	ERR12947594	CNS_G00305	yes
Aganisia cyanea (Lindl.) Rchb.f.	Zygopetalinae	Cymbidieae	Epidendroideae	Givnish *et al.* (2015)	-	yes
Agapanthus coddii F.M.Leight	-	-	Outgroup	NC 035971.1	-	yes
Agave americana L.	-	-	Outgroup	NC 032053.1	-	yes
Angraecum sesquipedale Thouars.	Angraecinae	Vandeae	Epidendroideae	Givnish *et al.* (2015)	-	yes
Anoectochilus emeiensis K.Y.Lang	Goodyerinae	Cranichideae	Orchidoideae	NC 033895.1	-	yes
Aphyllorchis montana Rchb.f	-	Neottieae	Epidendroideae	NC 030703.1	-	yes
Apostasia odorata Blume	-	-	Apostasioideae	NC 030722.1	-	yes
Apostasia shenzhenica Z.J.Liu & L.J.Chen	-	-	Apostasioideae	NC 039812.1	-	yes
Apostasia wallichii R.Br.	-	-	Apostasioideae	NC 036260.1	-	yes

frontiers

Arundina graminifolia (D.Don) Hochr.	Arethusinae	Arethuseae	Epidendroideae	MN171408.1	-	yes
Asparagus officinalis L.	-	-	Outgroup	NC 034777.1	-	yes
Batemannia colleyi Lindl.	Zygopetalinae	Cymbidieae	Epidendroideae	Serna-Sánchez *et al.* (2021)	-	yes
Bletilla striata Rchb.f.	Coelogyninae	Arethuseae	Epidendroideae	NC 028422.1	-	yes
Brachionidium cruziae L.O.Williams	Pleurothallidinae	Epidendreae	Epidendroideae	Serna-Sánchez *et al.* (2021)	-	yes
Bulbophyllum inconspicuum Maxim.	Dendrobiinae	Malaxideae	Epidendroideae	MN200377	-	yes
Calanthe aristulifera Rchb.f.	-	Collabieae	Epidendroideae	MN200378	-	yes
Calanthe sylvatica Lindl.	-	Collabieae	Epidendroideae	NC 044633.1	-	yes
Calopogon tuberosus (L.) Britton, Sterns & Poggenb.	Arethusinae	Arethuseae	Epidendroideae	Givnish *et al.* (2015)	-	yes
Calypso bulbosa var. *occidentalis* (Holz.) Cockerell.	Calypsoinae	Epidendreae	Epidendroideae	NC 040980.1	-	yes
Catasetum integerrimum Hook	Catasetinae	Cymbidieae	Epidendroideae	Givnish *et al.* (2015)	-	yes
Cattleya liliputana (Pabst) Van den Berg	Laeliinae	Epidendreae	Epidendroideae	NC 032083.1	-	yes
Cephalanthera damasonium Druce	-	Neottieae	Epidendroideae	NC 041179.1	-	yes
Cephalanthera rubra (L.) Rich.	-	Neottieae	Epidendroideae	NC 041181.1	-	yes
Changnienia amoena S.S.Chien.	Calypsoinae	Epidendreae	Epidendroideae	NC 045402.1	-	yes
Chaubardia surinamensis Rchb.f.	Zygopetalinae	Cymbidieae	Epidendroideae	Serna-Sánchez *et al.* (2021)	-	yes
Cheirostylis chinensis Rolfe	Goodyerinae	Cranichideae	Orchidoideae	MN641483.1	-	yes
Codonorchis lessonii Lindl.	-	Codonorchideae	Orchidoideae	Givnish *et al.* (2015)	-	yes
Coelogyne flaccida Lindl.	Coelogyninae	Arethuseae	Epidendroideae	Givnish *et al.* (2015)	-	yes
Corallorhiza striata Lindl.	Coelogyninae	Arethuseae	Epidendroideae	NC 040978.1	-	yes
Corallorhiza trifida Châtel	Coelogyninae	Arethuseae	Epidendroideae	NC 025662.1	-	yes
Coryanthes macrantha Hook	Stanhopeinae	Cymbidieae	Epidendroideae	Givnish *et al.*(2015)	-	yes
Cremastra appendiculata (D.Don) Makino	Coelogyninae	Arethuseae	Epidendroideae	NC 037439.1	-	yes
Cremastra unguiculata Finet.	Coelogyninae	Arethuseae	Epidendroideae	MN200381	-	yes
Cymbidium aloifolium (L.) Sw.	Cymbidiinae	Cymbidieae	Epidendroideae	NC 021429.1	-	yes
Cymbidium canaliculatum R.Br.	Cymbidiinae	Cymbidieae	Epidendroideae	ERR12947595	CNS_G00165	yes
Cymbidium ensifolium Sw.	Cymbidiinae	Cymbidieae	Epidendroideae	NC 028525.1	-	yes
Cymbidium faberi Rolfe	Cymbidiinae	Cymbidieae	Epidendroideae	NC 027743.1	-	yes
Cymbidium floribundum Lindl.	Cymbidiinae	Cymbidieae	Epidendroideae	MN173778.1	-	yes
Cymbidium lancifolium Hook	Cymbidiinae	Cymbidieae	Epidendroideae	NC 029712.1	-	yes

Cymbidium macrorhizon Lindl.	Cymbidiinae	Cymbidieae	Epidendroideae	NC 029713.1	-	yes
Cymbidium crassifolium Wall.	Cymbidiinae	Cymbidieae	Epidendroideae	NC 021433.1	-	yes
Cypripedium calceolus L.	-	-	Cypripedioideae	NC 045400.1	-	yes
Cypripedium japonicum Thunb.	-	-	Cypripedioideae	NC 027227.1	-	yes
Cyrtopodium flavum Link & Otto ex Rchb.	Cyrtopodiinae	Cymbidieae	Epidendroideae	Givnish *et al.* (2015)	-	yes
Dendrobium kingianum Bidwill ex Lindl.	Dendrobiinae	Malaxideae	Epidendroideae	LC331062.1	-	yes
Dendrobium moniliforme (L.) Sw.	Dendrobiinae	Malaxideae	Epidendroideae	MN200384	-	yes
Dichaea pendula (Aubl.) Cogn.	Zygopetalinae	Cymbidieae	Epidendroideae	Serna-Sánchez *et al.* (2021)	-	yes
Dipodium aff. *roseum** D.L.Jones & M.A.Clem.	Dipodiinae	Cymbidieae	Epidendroideae	ERR12947508	HTCG0828	no
Dipodium aff. *roseum** D.L.Jones & M.A.Clem.	Dipodiinae	Cymbidieae	Epidendroideae	ERR12947509	HTCG0830	no
Dipodium aff. *roseum** D.L.Jones & M.A.Clem.	Dipodiinae	Cymbidieae	Epidendroideae	ERR12947510	HTCG0831	no
Dipodium aff. *roseum** D.L.Jones & M.A.Clem.	Dipodiinae	Cymbidieae	Epidendroideae	ERR12947511	HTCG0832	yes
Dipodium aff. *stenocheilum** O.Schwarz	Dipodiinae	Cymbidieae	Epidendroideae	ERR12947512	HTCG1691	no
Dipodium ammolithum M.D.Barrett, R.L.Barrett & K.W.Dixon	Dipodiinae	Cymbidieae	Epidendroideae	ERR12947513	HTCG1372	yes
Dipodium atropurpureum D.L.Jones	Dipodiinae	Cymbidieae	Epidendroideae	ERR12947514	HTCG0760	yes
Dipodium atropurpureum D.L.Jones	Dipodiinae	Cymbidieae	Epidendroideae	ERR12947515	HTCG1679	no
Dipodium basalticum M.D.Barrett, R.L.Barrett & K.W.Dixon	Dipodiinae	Cymbidieae	Epidendroideae	ERR12947516	HTCG1693	yes
Dipodium campanulatum D.L.Jones	Dipodiinae	Cymbidieae	Epidendroideae	ERR12947517	HTCG1680	yes
Dipodium campanulatum D.L.Jones	Dipodiinae	Cymbidieae	Epidendroideae	ERR12947518	HTCG1681	no
Dipodium elegantulum D.L.Jones	Dipodiinae	Cymbidieae	Epidendroideae	ERR12947519	HTCG1682	yes
Dipodium ensifolium F.Muell.	Dipodiinae	Cymbidieae	Epidendroideae	ERR12947520	HTCG1343	yes
Dipodium hamiltonianum F.M.Bailey	Dipodiinae	Cymbidieae	Epidendroideae	ERR12947521	HTCG1683	yes
*Dipodium hamiltonianum** F.M.Bailey [syn. *Dipodium interaneum* D.L.Jones]	Dipodiinae	Cymbidieae	Epidendroideae	ERR12947522	HTCG0181	yes
Dipodium pandanum F.M.Bailey	Dipodiinae	Cymbidieae	Epidendroideae	ERR12947593	CNS_G01262	yes
Dipodium pandanum F.M.Bailey	Dipodiinae	Cymbidieae	Epidendroideae	ERR12947523	HTCG1694	no
Dipodium pardalinum D.L.Jones	Dipodiinae	Cymbidieae	Epidendroideae	ERR12947524	HTCG1684	yes
Dipodium pardalinum D.L.Jones	Dipodiinae	Cymbidieae	Epidendroideae	ERR12947525	HTCG1685	no
*Dipodium punctatum** (Sm.) R.Br. [syn. *Dipodium pulchellum* D.L.Jones & M.A.Clem.]	Dipodiinae	Cymbidieae	Epidendroideae	ERR12947526	HTCG1686	yes
Dipodium punctatum (Sm.) R.Br.	Dipodiinae	Cymbidieae	Epidendroideae	ERR12947527	HTCG0827	yes
Dipodium roseum D.L.Jones & M.A.Clem.	Dipodiinae	Cymbidieae	Epidendroideae	MN200386	-	no

Dipodium roseum D.L.Jones & M.A.Clem.	Dipodiinae	Cymbidieae	Epidendroideae	ERR12947528	HTCG1687	yes
Dipodium roseum D.L.Jones & M.A.Clem.	Dipodiinae	Cymbidieae	Epidendroideae	ERR12947529	HTCG1688	no
Dipodium stenocheilum O.Schwarz	Dipodiinae	Cymbidieae	Epidendroideae	ERR12947530	HTCG1689	no
Dipodium stenocheilum O.Schwarz	Dipodiinae	Cymbidieae	Epidendroideae	ERR12947531	HTCG1690	yes
Dipodium variegatum M.A.Clem. & D.L.Jones	Dipodiinae	Cymbidieae	Epidendroideae	ERR12947532	HTCG1692	yes
Elleanthus sodiroi Schltr.	-	Sobralieae	Epidendroideae	NC 027266.1	-	yes
Epipactis mairei Schltr.	-	Neottieae	Epidendroideae	NC 030705.1	-	yes
Epipactis thunbergia A.Gray.	-	Neottieae	Epidendroideae	MN200387	-	yes
Epipactis veratrifolia Boiss. & [illegible]	-	Neottieae	Epidendroideae	NC 030708.1	-	yes
Eria scabrilinguis Lindl.	-	Podochileae	Epidendroideae	MN477202.1	-	yes
Eulophia bicallosa (D.Don) P.F.Hunt & Summerh.	Eulophiinae	Cymbidieae	Epidendroideae	ERR12947533	HTCG1696	yes
Eulophia graminea Lindl.	Eulophiinae	Cymbidieae	Epidendroideae	ERR12947596	CNS_G02766	yes
Eulophia nuda Lindl.	Eulophiinae	Cymbidieae	Epidendroideae	ERR12947534	HTCG1697	yes
Eulophia petersii Rchb.f.	Eulophiinae	Cymbidieae	Epidendroideae	Givnish *et al.* (2015)	-	no
Evotella carnosa (Lindl.) J.C.Manning & Goldblatt	Coryciinae	Orchideae	Orchidoideae	Givnish *et al.* (2015)	-	yes
Gastrochilus japonicus Schltr.	Aeridinae	Vandeae	Epidendroideae	NC 035833.1	-	yes
Geodorum densiflorum (Lam.) Schltr.	Eulophiinae	Cymbidieae	Epidendroideae	ERR12947597	CNS_G01890	yes
Gongora pleiochroma Rchb.f.	Stanhopeinae	Cymbidieae	Epidendroideae	Givnish *et al.* (2015)	-	yes
Goodyera fumata Thwaites.	Goodyerinae	Cranichideae	Orchidoideae	NC 026773.1	-	yes
Goodyera schlechtendaliana Rchb.f.	Goodyerinae	Cranichideae	Orchidoideae	MK134679.1	-	yes
Guarianthe aurantiaca (Bateman) Dressler & W.E.Higgins.	Laeliinae	Epidendreae	Epidendroideae	Givnish *et al.* (2015)	-	yes
Gymnadenia conopsea (L.) R.Br.	Orchidinae	Orchideae	Orchidoideae	MN200391	-	yes
Habenaria arenaria Lindl.	Orchidinae	Orchideae	Orchidoideae	Givnish *et al.* (2015)	-	yes
Habenaria ciliolaris Kraenzl.	Orchidinae	Orchideae	Orchidoideae	MN495954.1	-	yes
Hexalectris warnockii Ames & Correll.	Laeliinae	Epidendreae	Epidendroideae	MH444822.1	-	yes
Holcoglossum lingulatum (Aver.) Aver.	Aeridinae	Vandeae	Epidendroideae	NC 041465.1	-	yes
Holcoglossum subulifolium (Rchb.f.) Christenson.	Aeridinae	Vandeae	Epidendroideae	NC 041519.1	-	yes
Huntleya meleagris Lindl.	Zygopetalinae	Cymbidieae	Epidendroideae	Serna-Sánchez *et al.* (2021)	-	yes
Iris sanguinea Hornem	-	-	Outgroup	NC 029227.1	-	yes
Lilium pensylvanicum Ker Gawl.	-	-	Outgroup	NC 043876.1	-	yes

Liparis auriculata Blume ex Miq.	Malaxidinae	Malaxideae	Epidendroideae	MN200365	-	yes
Ludisia discolor (Ker Gawl.) Blume	Goodyerinae	Cranichideae	Orchidoideae	NC 030540.1	-	yes
Masdevallia coccinea Linden ex Lindl.	Pleurothallidinae	Epidendreae	Epidendroideae	NC 026541.1	-	yes
Maxillaria nasuta Rchb.f.	Maxillariinae	Cymbidieae	Epidendroideae	Givnish *et al.* (2015)	-	yes
Maxillaria sanderiana Rchb.f. ex Sander	Maxillariinae	Cymbidieae	Epidendroideae	Givnish *et al.* (2015)	-	yes
Neottia listeroides Lindl.	-	Neottieae	Epidendroideae	NC_030713.1	-	no
Neottia ovata Bluff & Fingerh.	-	Neottieae	Epidendroideae	NC 030712.1	-	yes
Nervilia simplex (Spreng.) Schltr.	-	Nervilieae	Epidendroideae	Givnish *et al.* (2015)	-	no
Neuwiedia zollingeri var. *singapureana* (Wall. ex Baker) de Vogel	-	-	Apostasioideae	LC199503.1	-	yes
Oberonia japonica (Maxim.) Makino	Malaxidinae	Malaxideae	Epidendroideae	NC 035832.1	-	yes
Oeceoclades pelorica (D.L.Jones & M.A.Clem.) D.L.Jones & M.A.Clem.	Eulophiinae	Cymbidieae	Epidendroideae	ERR12947535	HTCG1695	yes
Oncidium sphacelatum Lindl.	Oncidiinae	Cymbidieae	Epidendroideae	NC 028148.1	-	yes
Ophrys fusca subsp. *iricolor** (Desf.) K.Richt.	Orchidinae	Orchideae	Orchidoideae	AP018716.1	-	yes
Ophrys sphegodes Mill.	Orchidinae	Orchideae	Orchidoideae	AP018717.1	-	yes
Otoglossum globuliferum (Kunth) N.H.Williams & M.W.Chase	Zygopetalinae	Cymbidieae	Epidendroideae	Serna-Sánchez *et al.* (2021)	-	yes
Otostylis brachystali Schltr.	Zygopetalinae	Cymbidieae	Epidendroideae	Serna-Sánchez *et al.* (2021)	-	yes
Pabstia jugosa (Lindl.) Garay.	Zygopetalinae	Cymbidieae	Epidendroideae	Serna-Sánchez *et al.* (2021)	-	yes
Palmorchis pabstii Veyret.	-	Neottieae	Epidendroideae	NC 041190.1	-	yes
Paphiopedilum armeniacum S.C.Chen & F.Y.Liu.	-	-	Cypripedioideae	NC 026779.1	-	yes
Paphiopedilum niveum (Rchb.f.) Stein	-	-	Cypripedioideae	NC 026776.1	-	yes
Pelatantheria scolopendrifolia (Makino) Aver.	Aeridinae	Vandeae	Epidendroideae	NC 035829.1	-	yes
Pescatoria wallisii Linden & Rchb.f.	Zygopetalinae	Cymbidieae	Epidendroideae	Serna-Sánchez *et al.* (2021)	-	yes
Phaius tankervilleae (Banks) Blume.	-	Collabieae	Epidendroideae	Givnish *et al.* (2015)	-	yes
Phalaenopsis japonica (Rchb.f.) Kocyan & Schuit.	Aeridinae	Vandeae	Epidendroideae	Givnish *et al.* (2015)	-	yes
Phalaenopsis pulcherrima (Lindl.) J.J.Sm.	Aeridinae	Vandeae	Epidendroideae	MG459020.1	-	yes
Phragmipedium longifolium (Rchb.f. & Warsz.) Rolfe	-	-	Cypripedioideae	NC 028149.1	-	yes
Platanthera mandarinorum Rchb.f.	Orchidinae	Orchideae	Orchidoideae	MN200370	-	yes
Platystele aurea Garay.	Pleurothallidinae	Epidendreae	Epidendroideae	Serna-Sánchez *et al.* (2021)	-	yes
Pleione formosana Hayata	Coelogyninae	Arethuseae	Epidendroideae	NC 042197.1	-	yes
Pogonia ophioglossoides (L.) Ker Gawl.	-	Pogonieae	Vanilloideae	Givnish *et al.* (2015)	-	yes

Ponerorchis gracilis (Blume) X.H.Jin, Schuit. & W.T.Jin	Orchidinae	Orchideae	Orchidoideae	MN200376	-	yes
Scaphosepalum antenniferum Rolfe.	Pleurothallidinae	Epidendreae	Epidendroideae	Serna-Sánchez *et al.* (2021)	-	yes
Sobralia mucronate Ames & C.Schweinf.	-	Sobralieae	Epidendroideae	Givnish *et al.* (2015)	-	yes
Spiranthes sinensis (Pers.) Ames	Spiranthinae	Cranichideae	Orchidoideae	MK936427.1	-	yes
Teagueia (Luer) Luer.	Pleurothallidinae	Epidendreae	Epidendroideae	Serna-Sánchez *et al.* (2021)	-	yes
Telipogon glicensteinii Dodson & R.Escobar	Oncidiinae	Cymbidieae	Epidendroideae	Givnish *et al.* (2015)	-	yes
Thelymitra cyanea (Lindl.) Benth.	Thelymitrinae	Diurideae	Orchidoideae	Givnish *et al.* (2015)	-	yes
Thrixspermum japonicum Rchb.f.	Aeridinae	Vandeae	Epidendroideae	NC 035831.2	-	yes
Triphora trianthophora (Sw.) Rydb.	Triphorinae	Triphoreae	Epidendroideae	Givnish *et al.* (2015)	-	no
Tropidia polystachya Ames.	-	Tropidieae	Epidendroideae	Givnish *et al.* (2015)	-	yes
Vanda brunnea Rchb.f.	Aeridinae	Vandeae	Epidendroideae	NC 041522.1	-	yes
Vanda falcata Beer.	Aeridinae	Vandeae	Epidendroideae	NC 036372.1	-	
Vanilla aphylla Blume	-	Vanilleae	Vanilloideae	NC 035320.1	-	yes
Vanilla planifolia Andrews	-	Vanilleae	Vanilloideae	NC 026778.1	-	yes
Vanilla pompona Schiede	-	Vanilleae	Vanilloideae	NC 036809.1	-	yes
Zootrophion hirtzii Luer.	Pleurothallidinae	Epidendreae	Epidendroideae	Serna-Sánchez *et al.* (2021)	-	yes
Zygopetalum triste Barb.Rodr.	Zygopetalinae	Cymbidieae	Epidendroideae	Serna-Sánchez *et al.* (2021)	-	yes

References

Givnish, T. J., Spalink, D., Ames, M., Lyon, S. P., Hunter, S. J., Zuluaga, A., et al. (2015). Orchid phylogenomics and multiple drivers of their extraordinary diversification. *Proc. R. Soc B* 282, 20151553. doi: 10.1098/rspb.2015.1553

Serna-Sánchez, M. A., Pérez-Escobar, O. A., Bogarın, D., Torres-Jimenez, M. F., Alvarez-Yela, A. C., Arcila-Galvis, J. E., et al. (2021). Plastid phylogenomics resolves ambiguous relationships within the orchid family and provides a solid timeframe for biogeography and macroevolution. *Sci. Rep.* 11, 6858. doi: 10.1038/s41598–021-83664–5
