# Supplementary material for: Plastid phylogenomics reveals evolutionary relationships in the mycoheterotrophic orchid genus Dipodium and provides insights into plastid gene degeneration
Source: Front Plant Sci. 2024 Jun 13;15:1388537. doi: 10.3389/fpls.2024.1388537 (PMC11210000; doi:10.3389/fpls.2024.1388537)
Supplement: Supplementary Material 2 — (A) Details of plastid loci included in alignment of ML-phylogenetic and divergence-time estimations. (B) Parsimony informative sites (Pi) for each plastid gene. [file DataSheet_2.pdf]

Supplementary Material 3

Plastid phylogenomics reveals evolutionary relationships in the mycoheterotrophic orchid genus *Dipodium* and provides insights into plastid gene degeneration

Stephanie Goedderz,[*], Mark A. Clements, Stephen J. Bent, James A. Nicholls, Vidushi S. Patel, Darren M. Crayn, Philipp M. Schlüter, Katharina Nargar[*]

*** Correspondence:**
Stephanie Goedderz: stephanie.goedderz@jcu.edu.au
Katharina Nargar: katharina.nargar@csiro.au

ML-Phylogenetic tree of Orchidaceae.

frontiers

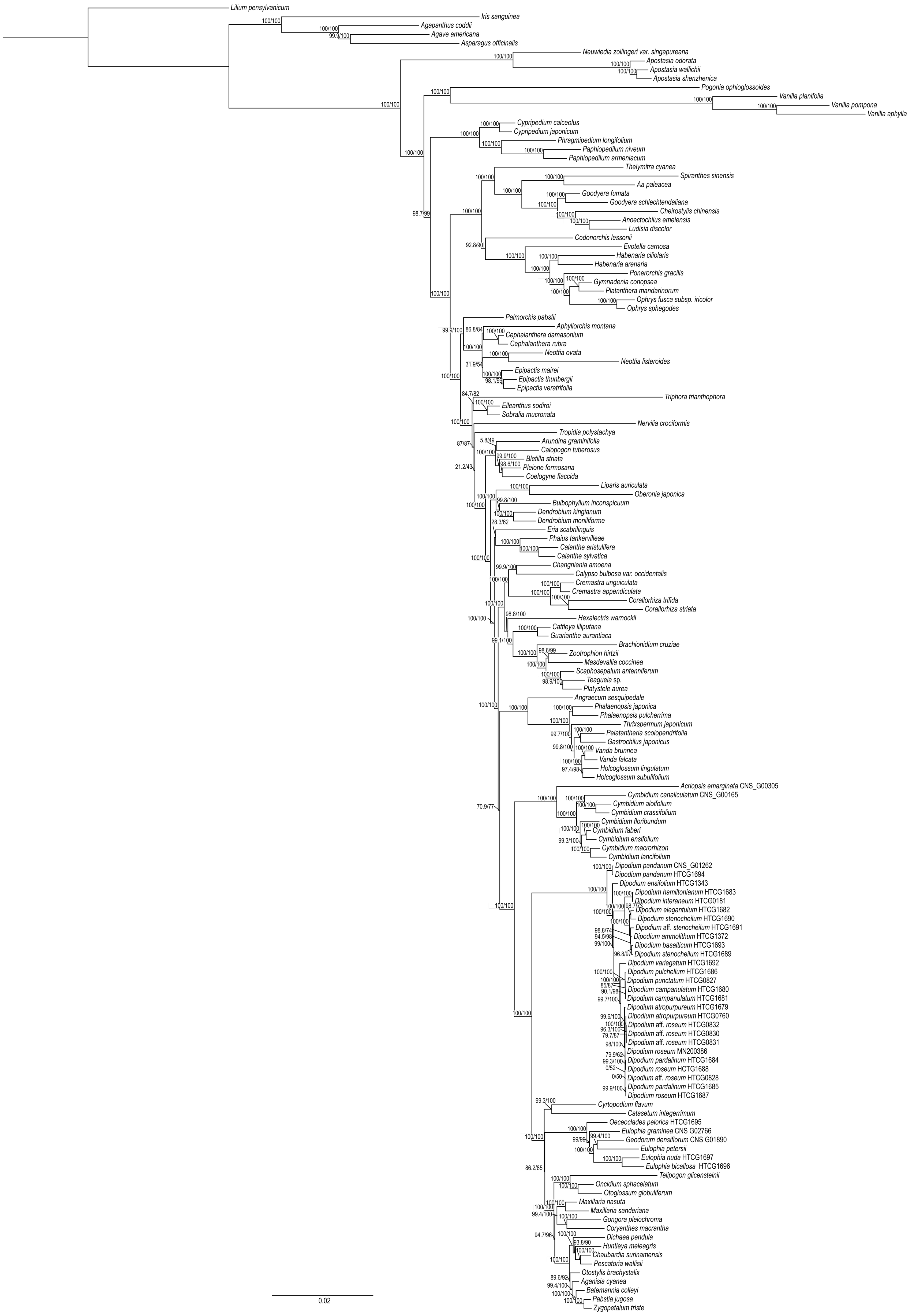

Figure S3.1: Phylogenetic relationships in Orchidaceae. Maximum likelihood tree based on 68 plastid loci and 148 taxa. Support values are given above each branch, SHaLRT is followed by UFBoot values. Scale bar represents branch length, along which 0.02 per-site substitutions are expected.
