# Supplementary material for: Plastid phylogenomics reveals evolutionary relationships in the mycoheterotrophic orchid genus Dipodium and provides insights into plastid gene degeneration
Source: Front Plant Sci. 2024 Jun 13;15:1388537. doi: 10.3389/fpls.2024.1388537 (PMC11210000; doi:10.3389/fpls.2024.1388537)
Supplement: Supplementary Material 3 — ML-Phylogenetic tree of Orchidaceae. [file DataSheet_3.pdf]

Supplementary Material 4

Plastid phylogenomics reveals evolutionary relationships in the mycoheterotrophic orchid genus *Dipodium* and provides insights into plastid gene degeneration

Stephanie Goedderz*, Mark A. Clements, Stephen J. Bent, James A. Nicholls, Vidushi S. Patel, Darren M. Crayn, Philipp M. Schlüter, Katharina Nargar*

*** Correspondence:**
Stephanie Goedderz: stephanie.goedderz@jcu.edu.au
Katharina Nargar: katharina.nargar@csiro.au

Table S4.1: Model Comparison by AICM (Akaike Information Criterion by MCMC), evaluated with the model selection criterion by AIC of Fabozzi et al., (2014). Best fit model for the data set after AICM is the optimised relaxed clock, birth-death model (in bold).

Model	AICM likelihood	+/-	burn-in	bootstrap replicates	delta AICM
**Optimised relaxed clock/Birth-death**	**432312.4507**	**2.5475**	**10**	**1000**	**0**
Optimised relaxed clock/ Yule	432319.0708	2.5574	10	1000	6.6201
Strict clock/ Birth-death	437771.8470	1.6389	10	1000	5459.3963
Strict clock/ Yule	437764.7433	1.5339	10	1000	5452.2926

From an article (appendix) titled: *Model Selection Criterion: AIC and BIC*:
The Basics of Financial Econometrics: Tools, Concepts, and Asset Management Applications. Frank J. Fabozzi, Sergio M. Focardi, Svetlozar T. Rachev and Bala G. Arshanapalli. © 2014 John Wiley & Sons, Inc. Published 2014 by John Wiley & Sons, Inc.

Table S4.2: Comparison divergence-time estimations of major Orchidaceae linages (subfamilies), the tribe Cymbidieae and subtribe Dipodiinae. Best fit model for the data set after AICM is the optimised relaxed clock, birth-death model (in bold). Absolute ages in 'million years ago' (Ma). MRCA: Most common recent ancestor; HDP: Highest posterior density interval.

	**relaxed clock, Birth-death**			relaxed clock, Yule		
MRCA	**mean ages**	**95% HDP lower bound**	**95% HDP upper bound**	mean ages	95% HDP lower bound	95% HDP upper bound
Orchidaceae	**126.35**	**100.56**	**155.60**	124.24	100.49	152.42
Apostasioideae	**101.96**	**98.01**	**105.82**	101.96	98.18	105.95
Vanilloideae	**93.26**	**89.27**	**97.05**	93.23	89.38	97.13
Cypripedioideae	**88.33**	**84.44**	**92.37**	88.30	84.33	92.37
Orchidoideae	**77.71**	**74.15**	**81.52**	77.71	73.83	81.22
Epidendroideae	**77.71**	**74.15**	**81.52**	77.71	73.83	81.22
Cymbidieae	**42.24**	**34.25**	**50.11**	42.44	34.81	50.18
Dipodiinae	**33.33**	**26.41**	**40.59**	33.60	26.46	40.47

	strict clock, Birth-death			strict clock, Yule		
MRCA	mean ages	95% HDP lower bound	95% HDP upper bound	mean ages	95% HDP lower bound	95% HDP upper bound
Orchidaceae	140.06	134.79	145.77	139.97	134.67	145.65
Apostasioideae	106.69	103.73	109.70	106.65	103.62	109.65
Vanilloideae	100.26	97.24	103.26	100.21	97.17	103.15
Cypripedioideae	80.44	77.69	83.24	80.48	77.69	83.28
Orchidoideae	71.20	68.86	73.65	71.24	68.84	73.62
Epidendroideae	71.20	68.86	73.65	71.24	68.84	73.62
Cymbidieae	44.80	42.52	46.52	44.92	42.83	46.48
Dipodiinae	36.51	34.81	38.20	36.57	34.92	38.30
