# Supplementary material for: Plastid phylogenomics reveals evolutionary relationships in the mycoheterotrophic orchid genus Dipodium and provides insights into plastid gene degeneration
Source: Front Plant Sci. 2024 Jun 13;15:1388537. doi: 10.3389/fpls.2024.1388537 (PMC11210000; doi:10.3389/fpls.2024.1388537)
Supplement: Supplementary Material 4 — (A) Model comparison by AICM (Akaike Information Criterion by MCMC). (B) Comparison divergence-time estimations of major Orchidaceae linages (subfamilies), the tribe Cymbidieae and subtribe Dipodiinae. [file DataSheet_4.pdf]

Supplementary Material 5

Plastid phylogenomics reveals evolutionary relationships in the mycoheterotrophic orchid genus *Dipodium* and provides insights into plastid gene degeneration

Stephanie Goedderz[*], Mark A. Clements, Stephen J. Bent, James A. Nicholls, Vidushi S. Patel, Darren M. Crayn, Philipp M. Schlüter, Katharina Nargar[*]

*** Correspondence:**
Stephanie Goedderz: stephanie.goedderz@jcu.edu.au
Katharina Nargar: katharina.nargar@csiro.au

Maximum-clade-credibility tree from Bayesian divergence-time estimations of Orchidaceae.

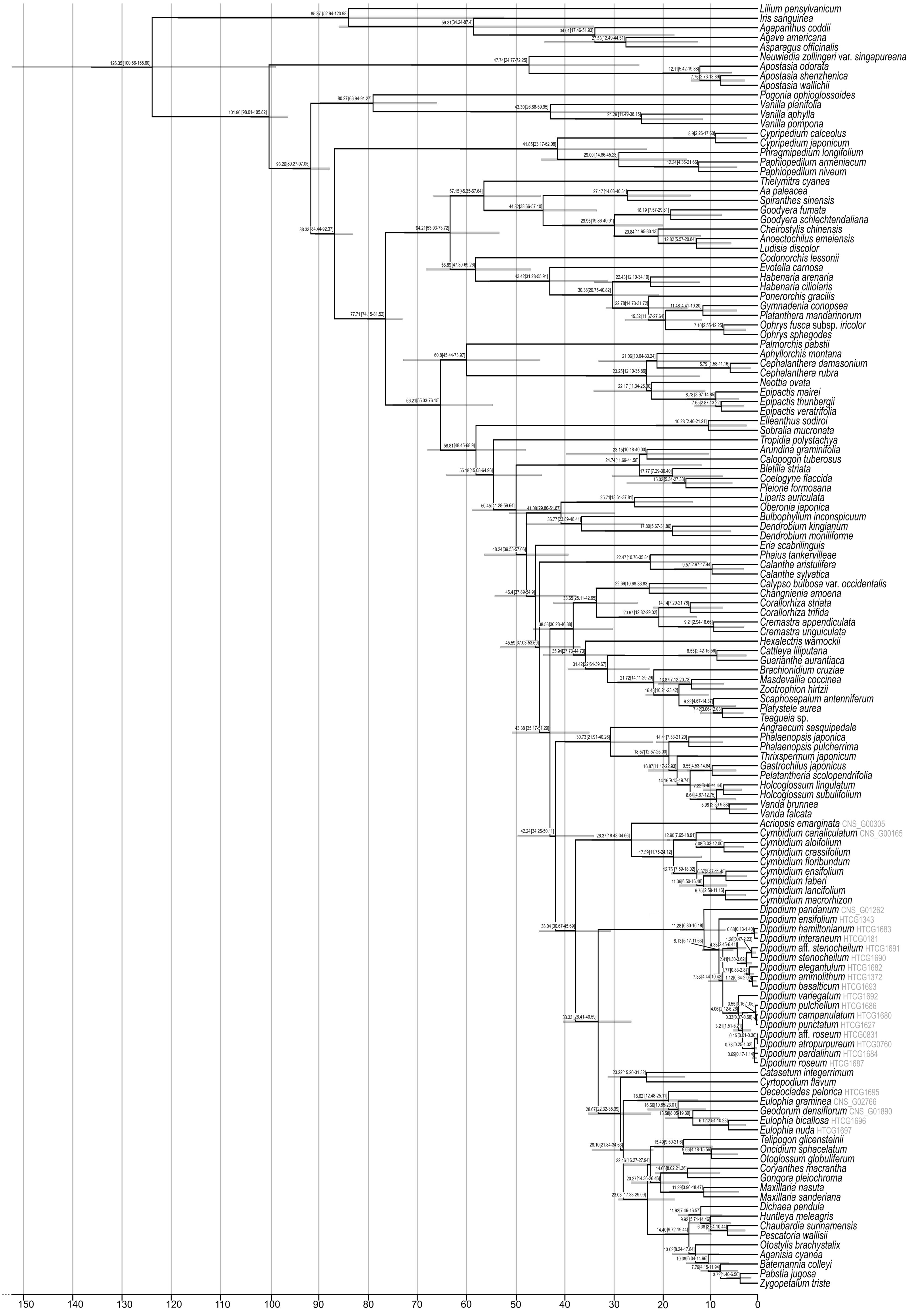

Figure S5.1: Chronogram of Orchidaceae. Maximum-clade-credibility tree from Bayesian divergence-time estimation in BEAST2 based on 30 plastid loci, 134T and an optimised relaxed molecular clock model under the birth-death prior. Divergence times (million years ago) are shown at each node, together with 95% highest posterior density (HDP) values indicated by grey bars and values in parentheses.
