# Supplementary material for: Plastid phylogenomics reveals evolutionary relationships in the mycoheterotrophic orchid genus Dipodium and provides insights into plastid gene degeneration
Source: Front Plant Sci. 2024 Jun 13;15:1388537. doi: 10.3389/fpls.2024.1388537 (PMC11210000; doi:10.3389/fpls.2024.1388537)
Supplement: Supplementary Material 5 — Maximum-clade-credibility tree from Bayesian divergence-time estimations of Orchidaceae. [file DataSheet_5.pdf]

Supplementary Material 6

Plastid phylogenomics reveals evolutionary relationships in the mycoheterotrophic orchid genus *Dipodium* and provides insights into plastid gene degeneration

Stephanie Goedderz*, Mark A. Clements, Stephen J. Bent, James A. Nicholls, Vidushi S. Patel, Darren M. Crayn, Philipp M. Schlüter, Katharina Nargar*

*** Correspondence:**
Stephanie Goedderz: stephanie.goedderz@jcu.edu.au
Katharina Nargar: katharina.nargar@csiro.au

Table S6.1: Summery of the reference-guided assembly features of 24 *Dipodium* plastomes reconstructed in this study.

	Samlpe	# trimmed reads	# mapped reads	mean coverage
1	*Dipodium ammolithum* HTCG1372	8,131,829	51,851	63
2	*Dipodium atropurpureum* HTCG0760	3,802,046	114,178	166
3	*Dipodium atropurpureum* HTCG1679	5,293,802	76,117	71
4	*Dipodium basalticum* HTCG1693	7,476,898	56,106	73
5	*Dipodium campanulatum* HTCG1680	4,928,715	33,297	31
6	*Dipodium campanulatum* HTCG1681	4,714,583	65,257	75
7	*Dipodium elegantulum* HTCG1682	5,696,744	40,461	48
8	*Dipodium ensifolium* HTCG1343	11,895,622	483,386	627
9	*Dipodium hamiltonianum* HTCG1683	11,327,386	54,724	63
10	*Dipodium interaneum* HTCG0181	3,709,623	53,624	75
11	*Dipodium pandanum* CNS_G01262	332,604	89,782	204
12	*Dipodium pardalinum* HTCG1684	8,086,850	70,697	67
13	*Dipodium pardalinum* HTCG1685	27,999,734	201,257	198
14	*Dipodium pulchellum* HTCG1686	6,795,229	85,282	93
15	*Dipodium punctatum* HTCG0827	8,987,460	90,899	120
16	*Dipodium roseum* HTCG1687	7,399,306	90,802	101
17	*Dipodium roseum* HTCG1688	6,207,464	52,233	50
18	*Dipodium* aff. *roseum* HTCG0828	7,953,368	251,830	353
19	*Dipodium* aff. *roseum* HTCG0830	4,347,368	56,127	74
20	*Dipodium* aff. *roseum* HTCG0831	11,388,084	357,584	475
21	*Dipodium* aff. *roseum* HTCG0832	10,706,617	244,503	336
22	*Dipodium stenocheilum* HTCG1689	4,657,080	38,117	44
23	*Dipodium stenocheilum* HTCG1690	6,026,488	37,602	47
24	*Dipodium variegatum* HTCG1692	4,256,510	41,813	44

Table S6.2: Comparison of the de novo assembled contigs/ plastomes using SPAdes 3.15 (Bankevich et al., 2012) and GetOrganelle v1.7.7.0 (Jin et al., 2020).

**Sample name**	**GetOrganelle # contigs**	**GetOrganelle total length [bp]**	**GetOrganelle longest contig [bp]**	**GetOrganelle >10kb [bp]**	**SPAdes #contigs**	**SPAdes total length [bp]**	**SPAdes longest contig [bp]**	**SPAdes >10kb [bp]**
Dipodium basalticum HTCG1693	28	108241	13089	10,972; 13,089	18	306064	41889	41889; 40051; 29646; 28302; 22640; 22065; 15996; 15808; 14491; 12338
Dipodium aff. *roseum* HTCG0828 (1)	40	58705	5569	0	17	305446	39361	39361; 27557; 23221; 22753; 22402; 20460; 20442; 18521; 17904; 17070
Dipodium aff. *roseum* HTCG0830 (2)	8	132923	53547	53427; 26121; 10599; 21193	13	265211	33140	33140; 31225; 31104; 23134; 21584; 20739; 20458; 20452; 18339; 17910
Dipodium aff. *roseum* HTCG0831 (3)	13	43008	3786	0	19	338511	57035	57035; 23060; 22753; 22369; 21584; 20469; 20460; 18397; 17910; 17070
Dipodium aff. roseum HTCG0832 (4)	36	37103	2318	0	18	332877	39684	39684; 39400; 22753; 22410; 21584; 20469; 20460; 18504; 17409; 17359
Dipodium ammolithum HTCG1372	6	140712	81300	81300; 21854; 16752; 10269	8	197021	81090	81090; 37009; 20633; 17961; 13643; 10059
Dipodium atropurpureum HTCG0760 (1)	complete plastome	141316	complete plastome	141316	18	272124	39684	39684; 30614; 22765; 21584; 20492; 17070; 16960; 14393; 13912; 12535
Dipodium atropurpureum HTCG1679 (2)	47	84434	10229	10229	10	168241	68294	68294; 22788; 13311; 12598; 12079; 10315
Dipodium campanulatum HTCG1680 (1)	9	145432	38520	38520; 30377; 16715; 14536; 12071; 10604	9	140791	43054	43054; 28351; 27754
Dipodium campanulatum HTCG1681 (2)	7	134128	44014	44014; 38521; 24561; 10604	7	137437	82450	82450; 16531; 10394
Dipodium elegantulum HTCG1682	4	150831	138555	138555	3	123154	67784	67784; 49042
Dipodium ensifolium HTCG1343	37	56060	3685	0	15	305519	38664	38664; 33336; 32359; 31601; 26839; 20536; 18720; 17566; 16943; 16591
Dipodium interaneum HTCG0181	6	127444	81192	81192; 31337	8	152820	70574	70574; 22365; 15180; 11904
Dipodium hamiltonianum HTCG1683	52	51631	3203	0	6	145596	81141	81141; 22366; 19288
Dipodium pandanum CNS_G01262	complete plastome	143979	complete plastome	143979	7	125920	55296	55296; 17440; 14330; 13191; 11102
Dipodium pardalinum HTCG1684 (1)	44	93561	7660	0	13	194321	54563	54563; 19255; 15339; 13815; 13806; 12546; 11985; 11063; 10489
Dipodium pardalinum HTCG1685 (2)	27	16042	1158	0	0	0		0

Dipodium pulchellum HTCG1686	20	120129	34366	19368; 19512; 34366	11	175871	38565	38565; 29858; 19264; 16360; 14588; 14401; 13030; 10366
Dipodium punctatum HTCG0827	26	108558	19272	19272; 14329; 10024	17	342074	68439	68439; 40469; 32514; 22763; 20393; 19103; 18534; 16540; 16485; 16439
Dipodium roseum HTCG1687 (1)	50	46137	3647	0	12	237983	47608	47608; 44249; 30891; 25429; 18214; 15651; 11923; 10366
Dipodium roseum HTCG1688 (2)	56	68870	4307	0	6	148369	97218	97218; 15046; 11293; 10367
Dipodium stenocheilum HTCG1689 (1)	11	129308	46908	46908; 32560; 12328; 10074	8	126692	48177	48177; 25386; 13993; 10269
Dipodium stenocheilum HTCG1690 (2)	3	150561	140419	140419	5	133489	61553	61553; 49257
Dipodium variegatum HTCG1692	37	110519	25631	25631	7	145577	64742	64742; 33447; 19561
