# Supplementary material for: Plastid phylogenomics reveals evolutionary relationships in the mycoheterotrophic orchid genus Dipodium and provides insights into plastid gene degeneration
Source: Front Plant Sci. 2024 Jun 13;15:1388537. doi: 10.3389/fpls.2024.1388537 (PMC11210000; doi:10.3389/fpls.2024.1388537)
Supplement: Supplementary Material 6 — (A) Summary of reference-guided assembly features of 24 newly generated Dipodium plastomes. (B) Comparison of the de novo assembled contigs/ plastomes using SPAdes 3.15 and GetOrganelle v1.7.7.0. [file DataSheet_6.pdf]

frontiers

Supplementary Material 7

Plastid phylogenomics reveals evolutionary relationships in the mycoheterotrophic orchid genus *Dipodium* and provides insights into plastid gene degeneration

Stephanie Goedderz*, Mark A. Clements, Stephen J. Bent, James A. Nicholls, Vidushi S. Patel, Darren M. Crayn, Philipp M. Schlüter, Katharina Nargar*

*** Correspondence:**
Stephanie Goedderz: stephanie.goedderz@jcu.edu.au
Katharina Nargar: katharina.nargar@csiro.au

Circular plastome maps of 24 newly generated *Dipodium* plastomes.

frontiers

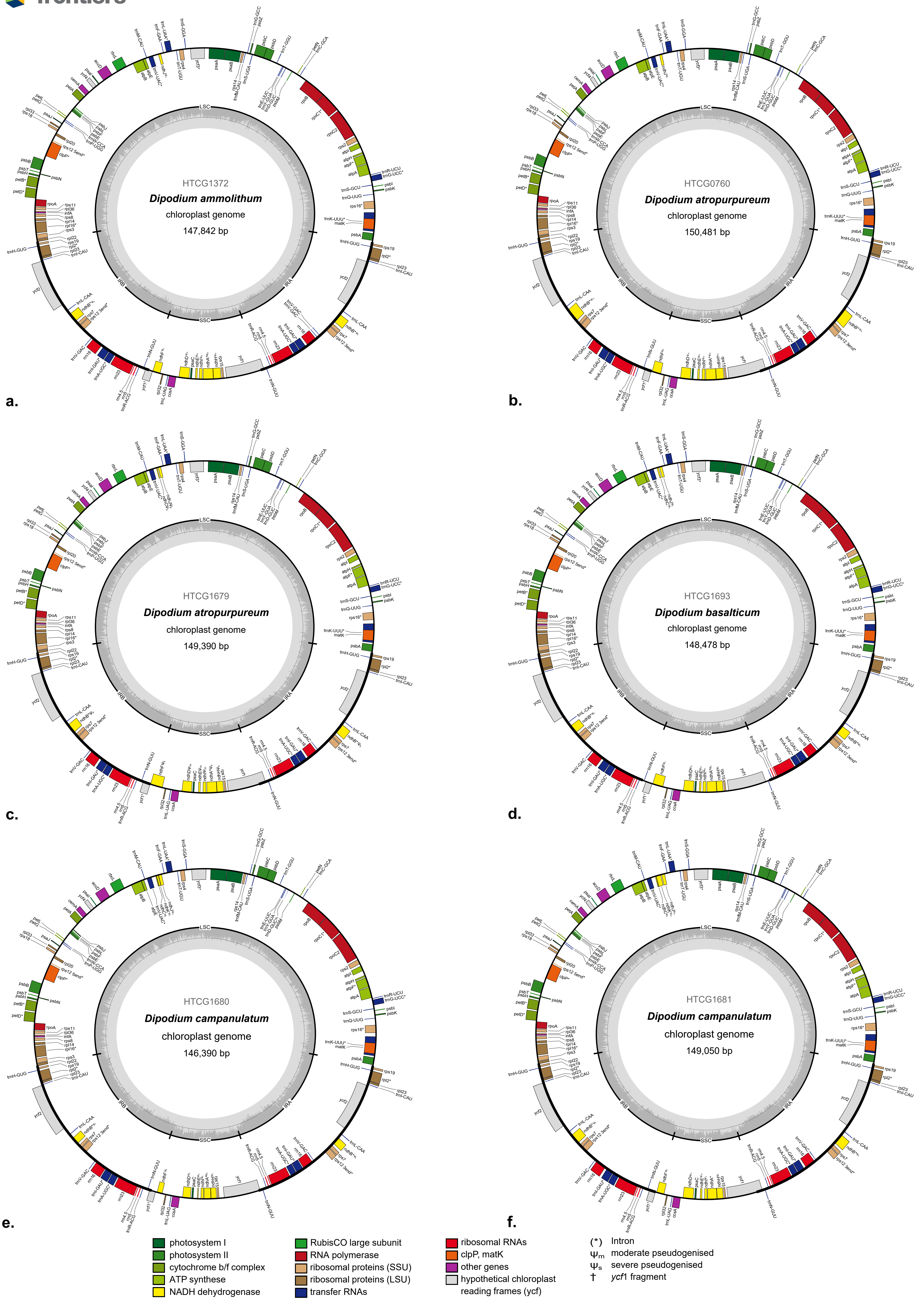

Figure S7.1: Circular plastome maps of a. *D. ammolithum* (HTCG1372), b., c. *D. atropurpureum* (HTCG0760, HTCG1679), d. *D. basalticum* (HTCG1683) and e., f. *D. campanulatum* (HTCG1680, HTCG1681). Genes outside the circle are transcribed in a clockwise direction, those inside the circle are transcribed in a counterclockwise direction. The dark grey inner circle corresponds to the G/C content, and the lighter grey to the A/C content. SSC: Small Single Copy; LSC: Large Single Copy: IRA/B: Inverted Repeat A/B.

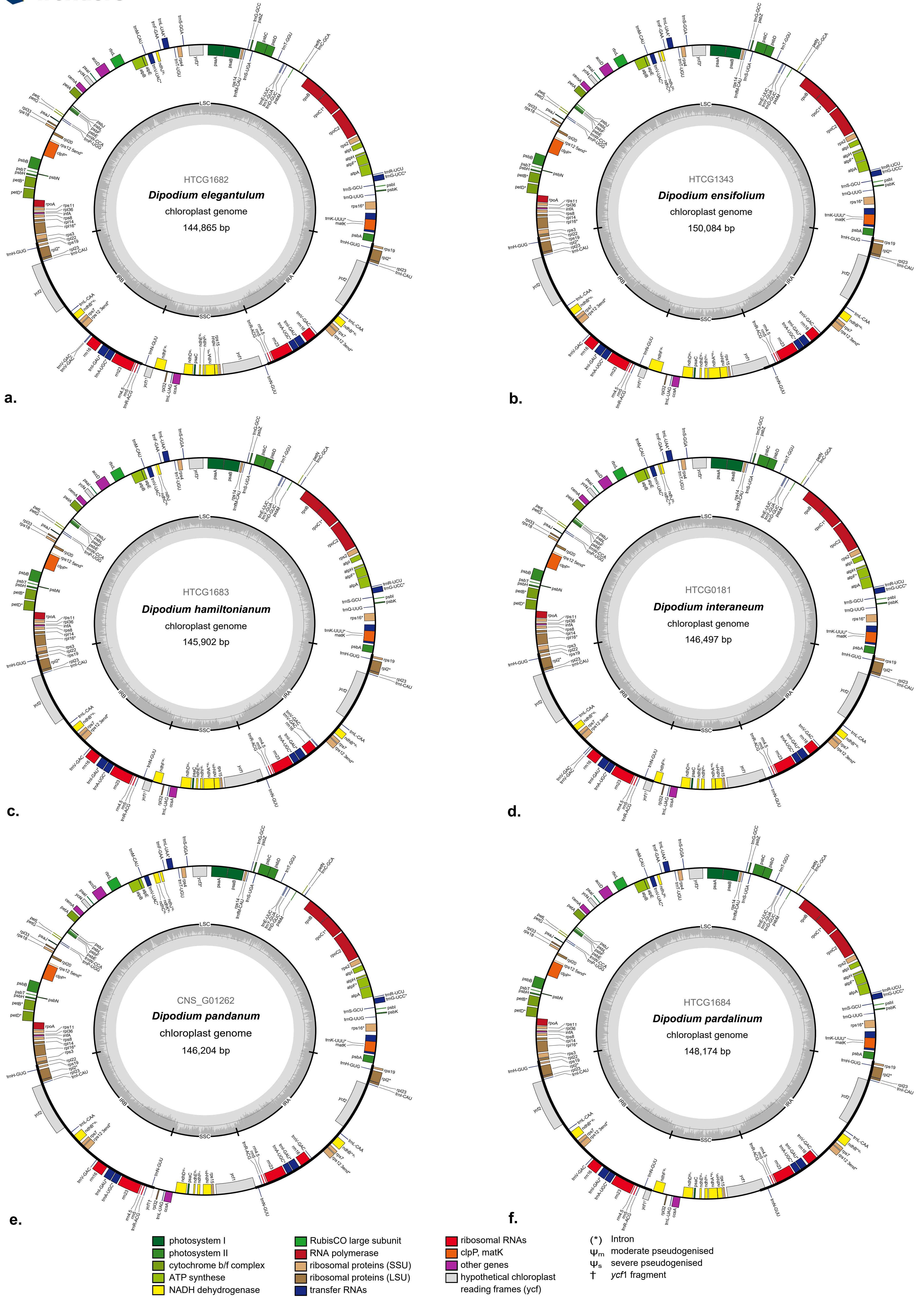

Figure S7.2: Circular plastome maps of a. *D. elegantulum* (HTCG1682), b. *D. ensifolium* (HTCG1343), c. *D. hamiltonianum* (HTCG1683), d. *D. interaneum* (HTCG0181), e. *D. pandanum* (CNS_G01262) and f. *D. pardalinum* (HTCG1684). Genes outside the circle are transcribed in a clockwise direction, those inside the circle are transcribed in a counterclockwise direction. The dark grey inner circle corresponds to the G/C content, and the lighter grey to the A/C content. SSC: Small Single Copy; LSC: Large Single Copy: IRA/B: Inverted Repeat A/B.

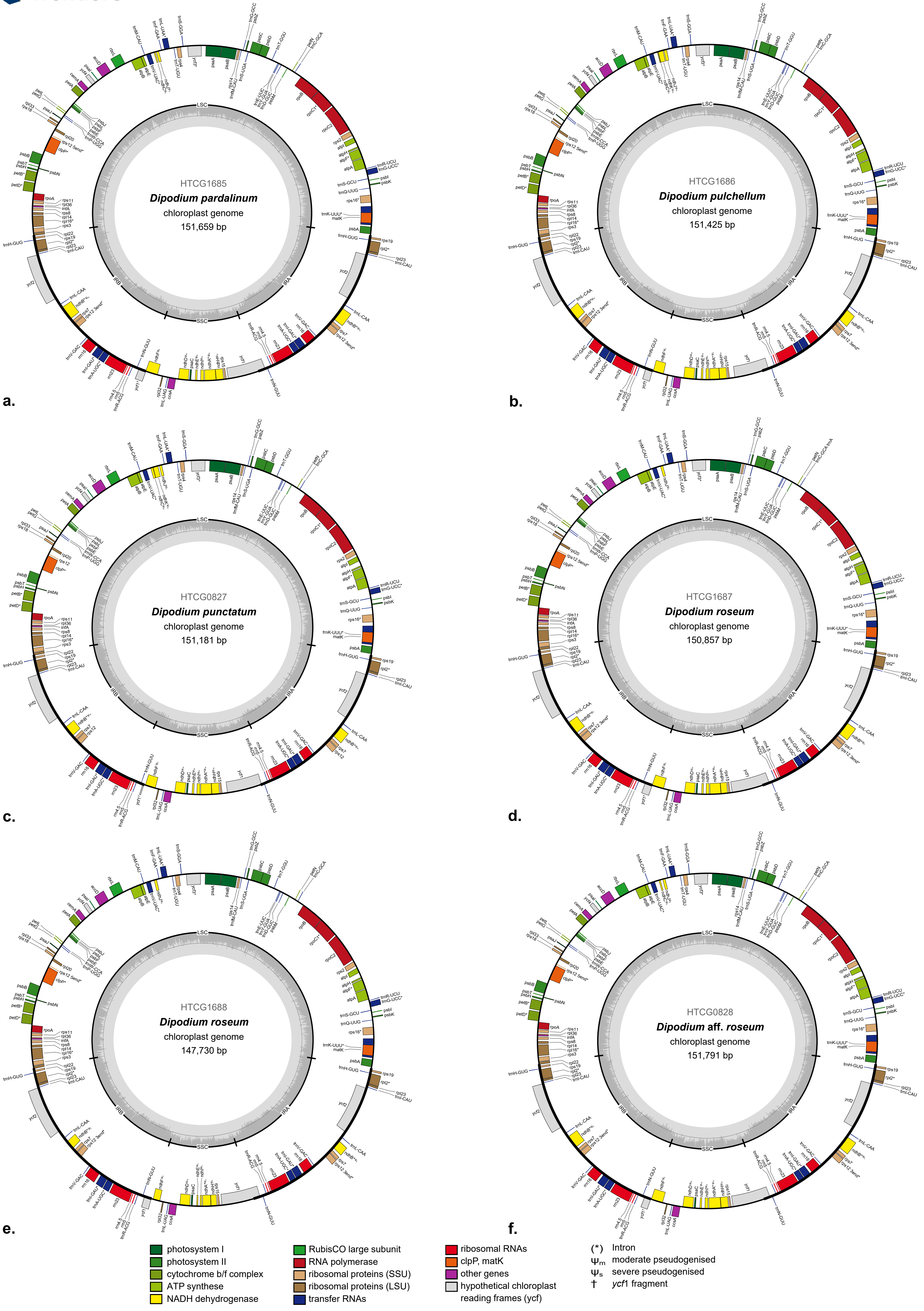

Figure S7.3: Circular plastome maps of a. *D. pardalinum* (HTCG1685), b. *D. pulchellum* (HTCG1686), c. *D. punctatum* (HTCG0827), d., e. *D. roseum* (HTCG1687, HTCG1688) and f. *D.* aff. *roseum* (HTCG0828). Genes outside the circle are transcribed in a clockwise direction, those inside the circle are transcribed in a counterclockwise direction. The dark grey inner circle corresponds to the G/C content, and the lighter grey to the A/C content. SSC: Small Single Copy; LSC: Large Single Copy: IRA/B: Inverted Repeat A/B.

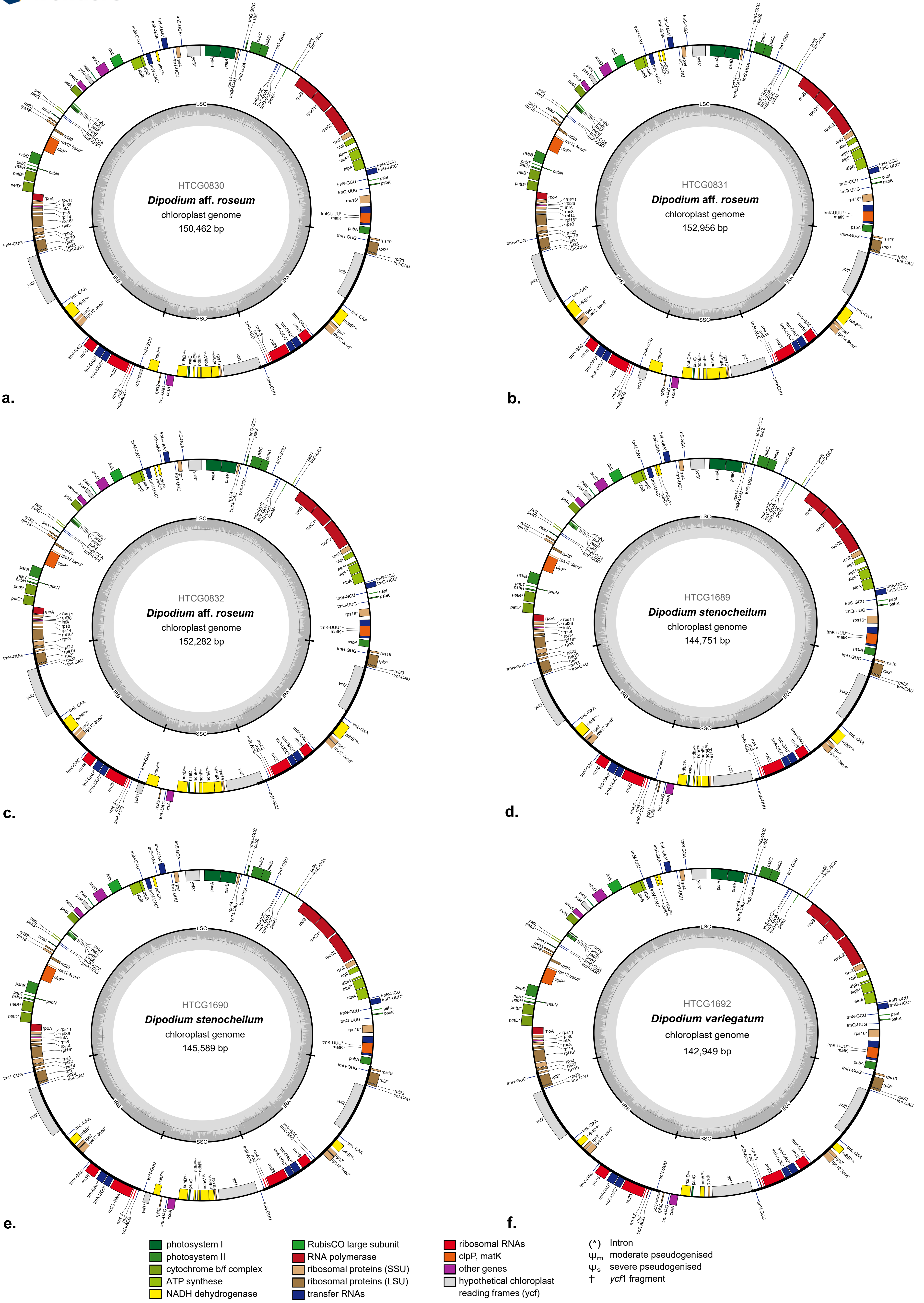

Figure S7.4: Circular plastome maps of a.-c. *D.* aff. *roseum* (HTCG0830, HTCG0831, HTCG0832), d., e. *D. stenocheilum* (HTCG1689, HTCG1690) and f. *D. variegatum* (HTCG1692) .Genes outside the circle are transcribed in a clockwise direction, those inside the circle are transcribed in a counterclockwise direction. The dark grey inner circle corresponds to the G/C content, and the lighter grey to the A/C content. SSC: Small Single Copy; LSC: Large Single Copy: IRA/ B: Inverted Repeat A/B.

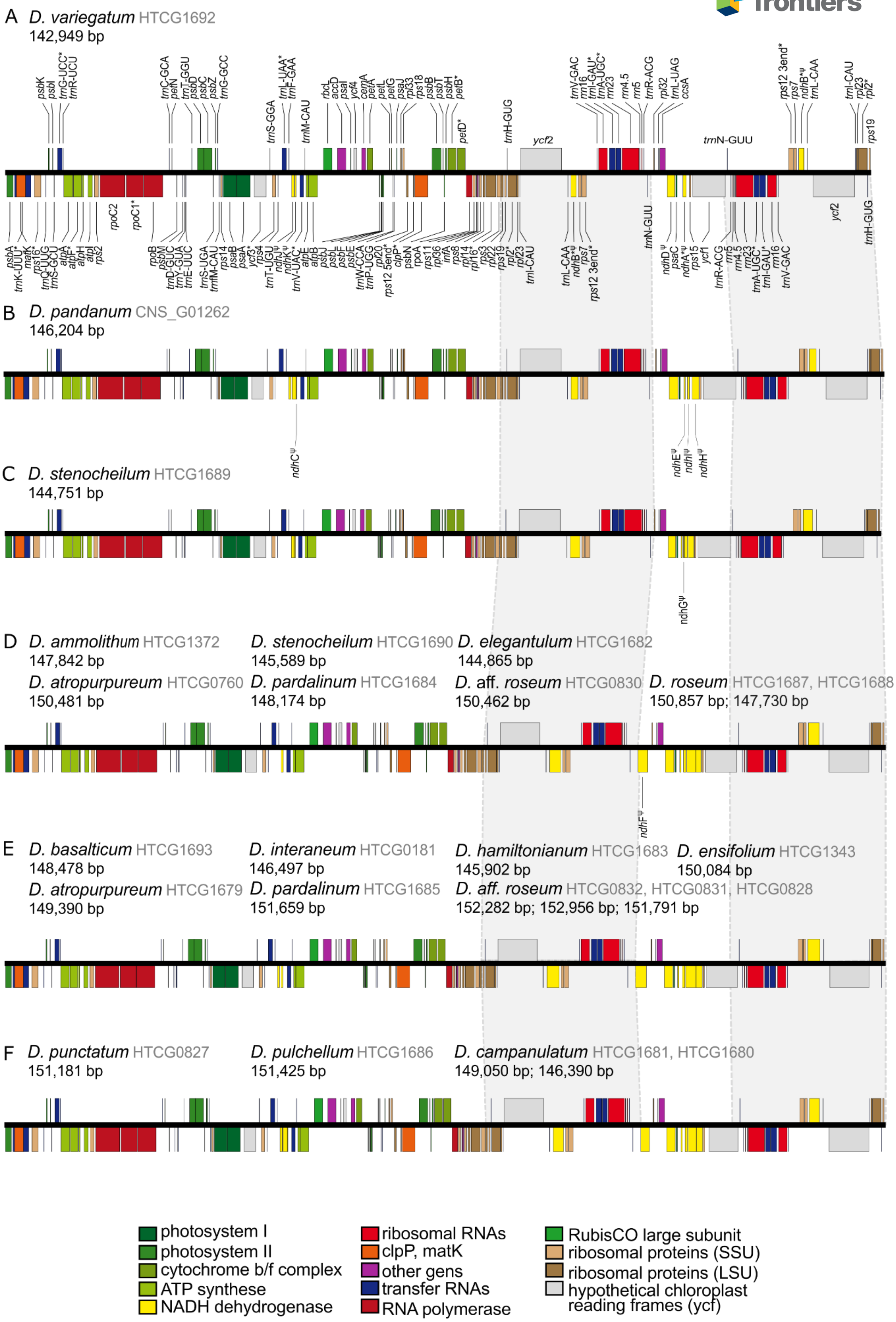

Figure S7.5: (A-F) Linear plastome maps of *Dipodium*. Genes drawn below the black line are transcribed clockwise, genes drawn above the black line are transcribed counterclockwise. Different functional groups of genes are colour-coded according to the legend. Phi (ψ) represents putative moderately or severely pseudogenised genes. Asterisks (*) represents intron containing genes. Grey shaded boxes highlighting IR regions.
